# Prognostic value of nucleotyping, DNA ploidy and stroma in high-risk stage II colon cancer

**DOI:** 10.1038/s41416-020-0974-8

**Published:** 2020-07-06

**Authors:** Lujing Yang, Pengju Chen, Li Zhang, Lin Wang, Tingting Sun, Lixin Zhou, Zhongwu Li, Aiwen Wu

**Affiliations:** 1 Key Laboratory of Carcinogenesis and Translational Research (Ministry of Education), Department of Pathology, Peking University Cancer Hospital & Institute, 100142 Beijing, People’s Republic of China; 2 Key Laboratory of Carcinogenesis and Translational Research (Ministry of Education), Department of Colorectal Surgery, Peking University Cancer Hospital & Institute, 100142 Beijing, People’s Republic of China

**Keywords:** Colorectal cancer, Colon cancer

## Abstract

**Background:**

Heterogeneity with respect to recurrence and survival in high-risk stage II colon cancer patients still exists, and further classification is urgently required. This study aimed to ascertain the prognostic value of DNA ploidy, stroma-tumour fraction and nucleotyping in the prognosis of high-risk stage II colon cancer.

**Methods:**

A total of 188 high-risk stage II colon cancer patients received radical surgery in Peking University Cancer Hospital, from 2009 to 2015. Status of mismatch repair proteins in tumours was analysed using immunohistochemistry. DNA ploidy, stroma-tumour fraction and nucleotyping were estimated by automated digital imaging systems.

**Results:**

Nucleotyping and DNA ploidy were significant prognostic factors, while stroma-tumour fraction were not significantly prognostic in the univariate analysis. In the multivariable model, the dominant contributory factor of disease-free survival was chromatin heterogeneous vs. chromatin homogeneous [HR 3.309 (95% CI: 1.668–6.564), *P* = 0.001].

**Conclusions:**

Our study indicates that nucleotyping is an independent prognostic factor in high-risk stage II colon cancer. Therefore, it may help subdivide patients into different subgroups and give them different strategies for follow-up and treatment in the future.

## Background

According to the recent statistical estimate of cancer prevalence in China, colorectal cancer (CRC) was the third most frequently diagnosed cancer in both males and females in 2011.^1^ Although some patients diagnosed with stage II colorectal cancer will recur and die of the disease, the need to administer postoperative adjuvant chemotherapy is disputed.^2-4^ At present, many experts do not recommend adjuvant chemotherapy to patients without one of the high-risk factors,^5,6^ which include lymph nodes sampling less than 12, poorly differentiated tumour, vascular or perineural invasion, pathological T4 stage tumours and clinical presentation with intestinal occlusion or perforation.^6^ Including one of these factors can be defined as high-risk stage II colon cancer.^6^ Although it is possible to categorise stage II colon cancer patients into low-risk group and high-risk group, previous studies have shown that there is heterogeneity within high-risk stage II colon cancer patients in terms of its prognosis.^7,8^ The present study investigates the use of additional biomarkers to further stratify patients with high-risk stage II colon cancer, providing a basis for individualised treatment in the future.

In recent years, several studies shown that chromosomal instability (CIN) is the driving force underlying cellular DNA aneuploidy in tumours.^9,10^ Errors in chromosome separation during cell division can result in an uneven distribution of chromosomes between the two daughter cells.^11^ This and replication stress may contribute to CIN.^11,12^ DNA aneuploidy or tetraploidy is an accepted marker of CIN that has been shown to be associated with poor prognosis in CRC.^13–15^ In addition, other studies have shown that CIN-associated cancers exhibit enhanced invasiveness, which may increase the likelihood of metastasis of CRC.^16^

Single nucleotide polymorphisms, chromosome structure and number changes, and genome arrangement may lead to genetic and epigenetic alterations in tumour cells,^17,18^ which can be detected by analysing chromatin texture in the nucleus.^19^ Chromatin texture analysis provides information on the spatial arrangement of pixel grayscale values in digital images of cell nuclei stained specifically for DNA.^19^ The nucleotyping method that we selected attempts to detect heterogeneous chromatin organisation through automatic texture analysis of digitised images of nuclei. A previous study has demonstrated that nucleotyping is a pan-cancer prognostic factor.^20^

The stroma-tumour fraction, described as the ratio of area occupied by carcinoma cells to total occupied by stromal cells and carcinoma cells in primary tumours, can be evaluated in sections stained with haematoxylin and eosin (H&E). Previous studies have shown that stroma-tumour fraction is an independent prognostic parameter in CRC and other solid epithelial tumours.^21–24^ In most of the studies, there is a 0.5 threshold for subdividing patients into low-stroma and high-stroma groups. Patients with high stroma were observed to have worse prognosis than patients with low stroma.^21-23^

Microsatellite instability can also cause CRC.^25^ The repair of microsatellite regions in DNA is controlled by mismatch repair (MMR) genes that encode various proteins, including MLH1, MSH2, MSH6 and PMS2. Deficiency of certain proteins may cause microsatellite instability, although patients with mismatch repair deficient (dMMR) CRC had improved disease-free survival (DFS) relative to patients with mismatch repair proficient status (pMMR).^26^

Currently, there are only few studies focused on high-risk stage II colon cancer patients. Therefore, the purpose of this study was to investigate the effect of DNA ploidy, stroma-tumour fraction and nucleotyping on the prognosis of high-risk stage II colon cancer.

## Methods

### Patient population

We retrospectively analysed all surgically resected high-risk stage II colon cancer samples in the Department of Pathology, Peking University Cancer Hospital, from 2009 to 2015. This study used formalin-fixed and paraffin-embedded (FFPE) samples of 188 recorded cases of high-risk stage II colon cancer without neoadjuvant chemoradiotherapy that were available for analyses.

### Immunohistochemistry

Immunohistochemistry (IHC) was performed using PowerVision Two-Step Histostaining Reagent (ImmunoVision Technologies, Brisbane, CA). Briefly, FFPE blocks were cut into 4-μm sections, dewaxed in xylene, rehydrated by decreasing alcohol gradient and washed twice in phosphate-buffered saline (PBS). Antigen retrieval was performed on tissue samples with EDTA buffer (pH 9.0; Santa Cruz Biochemistry, Dallas, TX) for 3 min in a pressure cooker. Endogenous peroxidase activity was blocked by incubation in 3% H_2_O_2_ solution at 25 °C for 10 min. After blocking with 5% normal goat serum, sections were incubated with mouse anti-MLH1 monoclonal antibody (Clone ES05, Shanghai GeneTech, Shanghai, China), anti-PMS2 monoclonal antibody (Clone EP51, Shanghai GeneTech, Shanghai, China), anti-MSH2 monoclonal antibody (Clone RED2, Shanghai GeneTech, Shanghai, China) and anti-MSH6 monoclonal antibody (EP49, Shanghai GeneTech, Shanghai, China) at 4 °C overnight. This was followed by incubation with anti-mouse Immunoglobulin-horseradish peroxidase conjugate (Beijing Zhongshan Golden Bridge Biotechnology, Beijing, China) at 25 °C for 30 min. Antibody binding was visualised using a 3,3′-diaminobenzidine kit (Beijing Zhongshan Golden Bridge Biotechnology, Beijing, China) according to the manufacturer’s instructions. For general negative controls, the primary antibodies were replaced by phosphate-buffered saline. All sections were examined microscopically and evaluated by two independent pathologists (Zhongwu Li and Li Zhang) who were unaware of the clinical information pertaining to the subjects. IHC staining was negative when all tumour cells showed loss of nuclear staining. Tumours that yielded negative staining results for at least one of the four MMR proteins, were classified as dMMR tumours, and all others were classified as pMMR tumours.

### Tumour sampling

For nucleotyping, DNA ploidy and stroma analyses, the pathologist selected one tumour block deemed representative from each patient and annotated the whole epithelial tumour region. Hence, no systematic selection was carried out. The DNA ploidy, stroma and nucleotyping analysis processes were carried out in Ningbo Meishan FTZ MBM Clinical Lab Co., Ltd.

### DNA image cytometry

FFPE tissue sections were cut at 5 μm and stained with H&E for defining the tumour region. One or two 50-μm sections, containing more than 90% representative tumour tissue, were cut from the tumour region marked on FFPE tissue blocks. The sections were deparaffinised in xylene, rehydrated through decreasing alcohol gradient, and washed twice in PBS. The sections were incubated at 37 °C at 83 × *g* for 1 h with 0.5 mg/mL protease VIII to disaggregate the cells. Cold PBS was added and the tubes were placed in an ice bath to stop enzymatic digestion. The cell suspension was filtered through a 60-μm mesh nylon filter and centrifuged at 415 × *g* (Sigma 3K-1) for 10 min. After discarding the supernatant, the pellet was resuspended in PBS. A volume of 100 μL of the solution was cytospinned at 250 × *g* for 5 min to prepare a monolayer of nuclei on a slide. The monolayer preparations were air-dried and fixed overnight in 4% formaldehyde before stained using Feulgen’s method.^27^

### Measurement of DNA content

The Feulgen-stained nuclei were measured with DNA Ploidy Working Station (Room 4, Kent, UK), as previous report.^27^ Briefly, an image of each nucleus was captured by a high-resolution digital scanner (Aperio AT2, Leica, Germany), and images were automatically grouped into different galleries for tumour nuclei, reference nuclei and discarded nuclei. DNA ploidy histograms were created from the integrated optical density (IOD) of the nuclei using PWS Classifier (Room 4, Kent, UK). The reference nuclei were used as an internal diploid control, and DNA ploidy histograms were classified into four groups: diploid, aneuploid, tetraploid and polyploid according to a previous report.^28^ Aneuploid, tetraploid and polyploid samples were grouped as non-diploid in this study.

### Nuclear texture analysis

Nucleotyping was automatically assessed as proposed in a previous study.^20^ Each tumour sample was independently classified using PWS Classifier and the same set of images of tumour nuclei that the DNA ploidy histogram was based on. Chromatin organisation was quantified by computing the entropy of pixel grey levels in a subregion of a nucleus. The frequency in which each pair of entropy and centre grey level occur throughout a nucleus was stored in a two-way table, known as the grey level entropy matrix (GLEM). GLEMs stratified on nuclear area and subregion size were concatenated to form a four-dimensional expansion of the GLEM called GLEM4D. In a previous study,^20^ an adaptive machine learning algorithm was applied to quantify the association between each element of the GLEM4D and the outcome of the patient. In the current study, these pretrained weights were directly applied to predict the outcome of a patient on the basis of the GLEM4D representation of its tumour. This was done by multiplying each element of the patient’s GLEM4D with the corresponding weight computed in the previous study,^20^ thereafter adding the products. The result was a continuous value termed the chromatin value, which describes the overall amount of chromatin disorder in a given patient sample.^20^ According to the threshold of 0.044, the tumours were classified into chromatin homogeneous (CHO, ≥ 0.044) or chromatin heterogeneous (CHE, < 0.044).

### Stroma-tumour fraction

Stroma-tumour fraction was determined on H&E stained histological sections. H&E stained sections were routinely estimated under 10 × 10 lens microscope to select the sections rich in tumour (tumour tissue > 50%, necrotic tissue < 10%). The stroma-tumour fraction was measured by Stroma Analyzer (Room 4, Kent, UK), as was described by Danielsen et al. ^29^ Briefly, the whole slides images of H&E stained sections were scanned with an Aperio AT2 digital slide scanner at ×40 (Leica, Germany), giving a resolution of 0.23 µm per pixel. Images with a resolution of 1.82 µm per pixel were used for image processing. A senior pathologist (Li Zhang) marked the tumour areas on the scanned images by using the software tool (Stroma analyzer, Room 4, Kent, UK). The stroma fraction in the selected tumour region was automatically calculated by the software (Stroma analyzer, Room 4, Kent, UK). Tumours with stroma fraction less than or equal to 0.50 were labelled low stroma, while those with stroma fraction greater than 0.50 were labelled high stroma.

### Follow-up

Patients were followed at 6 months intervals for the first 2 years after treatment, and annually thereafter. Evaluations consisted of physical examination, a complete blood count, serum carcino-embryonic antigen levels and blood chemical analysis. Proctoscopy, CT imaging of the abdomen and pelvis, and chest radiography were also routinely performed every 6–12 months after treatment.

### Statistical analysis

The endpoints were overall survival (OS) and DFS. OS was defined as the time between the date of initial surgery and date of death for any reason or the date of the last follow-up. DFS was defined as the time from the date of initial surgery to the date of death for any cause or the first local recurrence or metastasis. IBM SPSS Statistics for Macintosh, Version 20.0 (IBM Corp, Armonk, NY) software was used for all analyses. Kaplan–Meier survival curves with log-rank estimates were used to depict time-to-event parameters. Univariate and multivariate cox proportional hazards were established to obtained hazard ratios with 95% confidence interval for parameters. Correlation analysis were performed using Spearman correlation coefficients, >0 indicates a positive correlation and coefficient <0 indicates a negative correlation. The statistical significance level was set at 0.05. We established a total of six multivariable models, a correction for multiple comparisons was performed. Thus, a two-sided *P*-value of less than 0.008 was considered statistically significant for each multivariable model.

## Results

### Patient demography

Totally, 188 consecutive cases including 121 male and 67 female patients were involved in this study. The median age of patients was 62.2 years, the majority of patients was pT3 (pT3 vs. pT4, 66.5% vs. 33.5%) and 56.9% of all patients were treated with chemotherapy after surgery (Capecitabine alone, Oxaliplatin, leucovorin and 5-FU, or Oxaliplatin and Capecitabine). Other patients’ characteristic and distribution of relevant parameters are listed in Table 1.

**Table 1:** Distribution of relevant parameters.

Variables	*n*	%
Age, years		
≤63	90	47.9
>63	98	52.1
Gender		
Male	121	64.4
Female	67	35.6
Lymph nodes sampling		
≥12	152	80.9
<12	36	19.1
Histological grade		
Well differentiated	7	3.7
Moderately differentiated	123	65.4
Poorly differentiated	55	29.3
Mucinous differentiated	3	1.6
Vascular or perineural invasion		
Yes	55	29.3
No	133	70.7
pTstage		
pT3	125	66.5
pT4	63	33.5
Intestinal occlusion or perforation		
Yes	37	19.7
No	151	80.3
Mismatch repair status		
pMMR	124	66.0
dMMR	64	34.0
Adjuvant chemotherapy		
No	81	43.1
Yes	107	56.9
DNA ploidy		
Diploid	88	46.8
Non-diploid	100	53.2
Stroma		
Low stroma	153	81.4
High stroma	35	18.6
Nucleotyping		
Chromatin homogeneous	152	80.9
Chromatin heterogeneous	36	19.1

*pMMR* mismatch repair proficient, *dMMR* mismatch repair deficient.

At the end of follow-up, 161 patients were still alive and 35 patients had a recurrence or metastasis. Median OS and median DFS were 68 months (25–75% quartiles: 55–92 months) and 66 months (25–75% quartiles: 52–91 months), respectively.

A positive correlation between pathological T-stage and stroma-tumour fraction was observed in our cohort (coefficient = 0.153, *P* = 0.037), which meant that the higher the pathological T-stage, the more the tumour stroma. A negative correlation between diploid and poorly differentiated was observed (ρ = −0.215, *P* = 0.005). A positive correlation between DNA ploidy and nucleotyping was observed (ρ = 0.743, *P* < 0.001). Except for one diploid tumour, chromatin heterogeneous (CHE) was almost exclusively found in non-diploid tumours. A negative correlation between diploid and pMMR was observed (coefficient = −0.237, *P* = 0.001). (Supplementary Tables 1 and 3).

### Univariate prognostic factors

**Fig. 1:**
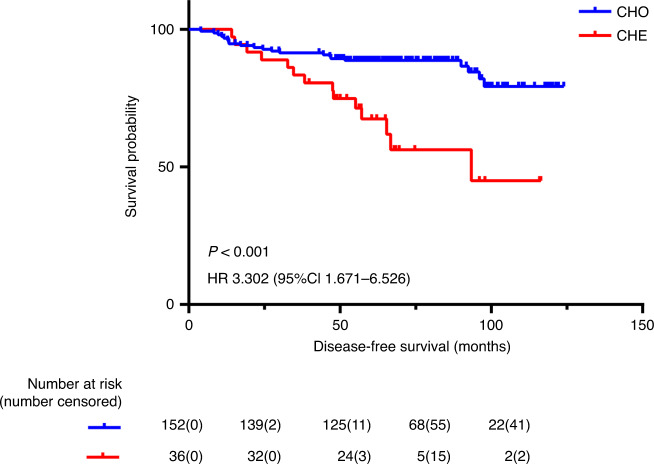
DFS in nucleotyping. Kaplan–Meier plots illustrating DFS for patients with tumours that were chromatin homogeneous (CHO) and chromatin heterogeneous (CHE) among patients with high-risk stage II colon cancer.

Of all 188 cases, our mean follow-up was 71 months. Analysis of DFS and OS in high-risk stage II colon cancer are shown in Table 2. DNA ploidy and nucleotyping were found to be significant in univariate analysis of DFS (*P* = 0.006 and *P* < 0.001, respectively) (Fig. 1). Patients with pT4 tumours had an inferior DFS compared to patients with pT3 tumours (*P* = 0.024). Stroma-tumour fraction was not a significant prognostic factor for DFS. In our cohort, 83.7% and 71.4% was the recurrence and metastasis rate of the stroma-low and stroma-high group, respectively, and the power calculation was 0.184. No significant prognostic impact was observed for dMMR, utilisation of adjuvant chemotherapy and other risk factors for high-risk stage II colon cancer in univariate analyses.

**Table 2:** Univariate analysis on overall survival and disease-free survival of the prognostic factors in high-risk stage II patients.

Variable	*n*	OS, %	HR (95%CI)	*P*-value	DFS, %	HR (95%CI)	*P*-value
Age				0.034			0.057
** ≤**63	90	80.0	1		75.6	1	
>63	98	90.8	0.431 (0.194–0.960)		86.7	0.520 (0.262–1032)	
Lymph nodes sampling				0.950			0.814
≥12	152	85.5	1		80.9	1	
<12	36	86.1	1.032 (0.390–2.727)		83.3	0.900 (0.373–2.169)	
Histological grade				0.690			0.433
Well differentiated	7	71.4	1		71.4	1	
Moderately differentiated	123	85.4	0.548 (0.127–2.375)		78.9	0.782 (0.185–3.310)	
Poorly differentiated	55	87.3	0.464 (0.096–2.248)		87.3	0.446 (0.092–2.156)	
Mucinous	3	100.0	–		100.0	–	
Vascular or perineural invasion				0.920			0.420
Yes	55	87.3	1		80.0	1	
No	133	85.0	0.956 (0.402–2.276)		82.0	0.745 (0.363–1.528)	
pTstage				0.053			0.024
pT3	125	90.4	1		87.2	1	
pT4	63	76.2	2.111 (0.974–4.576)		79.8	2.111 (0.974–4.576)	
Intestinal occlusion or perforation				0.349			0.574
Yes	37	81.1	1		78.4	1	
No	151	86.8	0.665 (0.281–1.573)		82.1	0.798 (0.362–1.757)	
Mismatch repair status				0.248			0.340
pMMR	124	84.7	1		80.6	1	
dMMR	64	87.5	0.611 (0.165–1.410)		82.8	0.704 (0.342–1.447)	
Adjuvant chemotherapy				0.122			0.244
No	81	81.5	1		77.8	1	
Yes	107	88.8	0.552 (0.258–1.184)		84.1	0.675 (0.347–1.313)	
DNA ploidy				0.053			0.006
Diploid	88	90.9	1		89.8	1	
Non-diploid	100	81.0	2.216 (0.969–5.068)		74.0	2.770 (1.296–5.918)	
Stroma				0.345			0.118
Low stroma	153	86.9	1		83.7	1	
High stroma	35	80.0	1.510 (0.638–3.573)		71.4	1.782 (0.855–3.711)	
Nucleotyping				0.001			<0.001
Chromatin homogeneous	152	89.5	1		86.2	1	
Chromatin heterogeneous	36	69.4	3.518 (1.618–7.646)		61.1	3.302 (1.671–6.526)	
DNA ploidy and stroma				0.135			0.011
Diploid and low stroma	77	90.9	1		89.6	1	
Diploid and high stroma or non-diploid and low stroma	87	83.9	1.802 (0.727–4.466)		79.3	2.081 (0.905–4.788)	
Non-diploid and high stroma	24	75.0	2.926 (0.982–8.712)		62.5	4.036 (1.556–10.472)	
Nucleotyping and stroma				0.033			0.003
Chromatin homogeneous and low-stroma	130	90.0	1		86.9	1	
Chromatin homogeneous and high-stroma or Chromatin heterogeneous and low-stroma	45	77.8	2.345 (1.028–5.349)		73.3	2.148 (1.026–4.499)	
Chromatin heterogeneous and high-stroma	13	69.2	3.575 (1.159–11.033)		53.8	4.161 (1.635–10.595)	
DNA ploidy and nucleotyping				0.002			<0.001
Diploid and chromatin homogeneous	87	90.8	1		89.7	1	
Diploid and chromatin heterogeneous or non-diploid and chromatin homogeneous	66	87.9	1.330 (0.499–3.545)		81.8	1.845 (0.777–4.382)	
Non-diploid and chromatin heterogeneous	35	68.6	4.140 (1.653–10.370)		60.0	4.632 (1.995–10.752)	

*pMMR* mismatch repair proficient, *dMMR* mismatch repair deficient, *OS* overall survival, *DFS* disease-free survival.

The combinations of nucleotyping and stroma; DNA ploidy and stroma and nucleotyping and DNA ploidy were analysed.

**Fig. 2:**
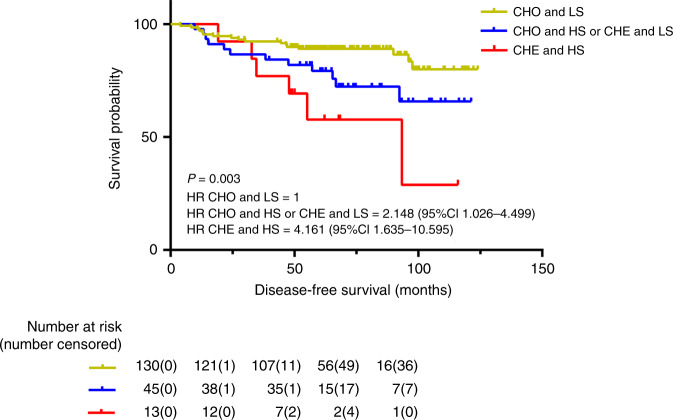
DFS in the combination of nucleotyping and stroma. Kaplan–Meier plots illustrating DFS for patients with tumours that were chromatin homogeneous and low stroma (CHO and LS), chromatin homogeneous and high stroma or chromatin heterogeneous and low stroma (CHO and HS or CHE and LS), and chromatin heterogeneous and high stroma (CHE and HS) among patients with high-risk stage II colon cancer.

According to the length of DFS, the combination of nucleotyping and stroma was divided into three groups, and it was found that chromatin homogeneous (CHO) and low-stroma patients had the highest 5-year DFS [89.2% (95% CI: 83.8–94.6%)] of the three groups (low risk), while the CHO and high-stroma or CHE and low-stroma patients had intermediate 5-year DFS [82.2% (95% CI: 70.6–93.8%)], referred to as medium-risk group. The HR was 2.148 (95% CI: 1.026–4.499). The CHE and high-stroma patients had the lowest 5-year DFS [61.5% (95% CI: 30.9–92.1%)], which is referred to as the high-risk group. The HR was 4.161 (95% CI: 1.635–10.595). The combination of nucleotyping and stroma was statistically significant on DFS (*P* = 0.003) (Fig. 2).

Similarly, as in a previous study,^29^ the combination of DNA ploidy and stroma was divided into three groups, which were respectively called low-risk, medium-risk and high-risk groups according to DFS. Low-stroma and diploid tumours represented patient groups with high 5-year DFS [89.6% (95% CI: 82.7–96.6%)], which we defined as low-risk group, while high-stroma and diploid tumours together with low-stroma and non-diploid tumours represented intermediate-risk group. Compared to the low-risk group, this group had decreased 5-year DFS [86.2% (95% CI: 78.8–93.6%)], which was defined as the medium-risk group. The HR was 2.081 (95% CI: 0.905–4.788). High-stroma and non-diploid tumours characterised a group of patients with low 5-year DFS [70.8% (95% CI: 51.2–90.4%)], which was defined as the high-risk group. The HR was 4.036 (95% CI: 1.556–10.472). The combination of DNA ploidy and stroma was statistically significant for DFS (*P* = 0.011).

**Fig. 3:**
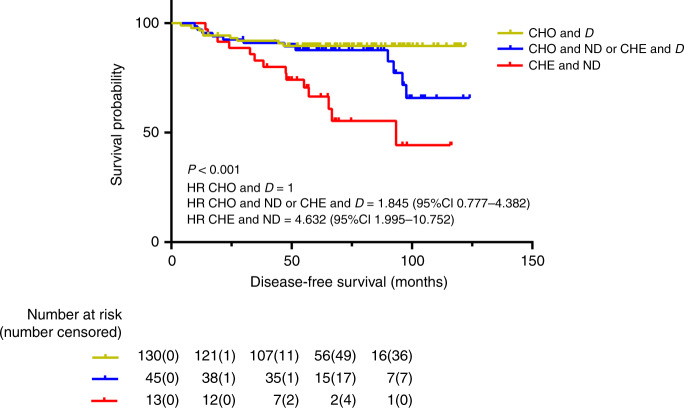
DFS in the combination of nucleotyping and DNA ploidy. Kaplan–Meier plots illustrating DFS for patients with tumours that were chromatin homogeneous and diploid (CHO and D), chromatin homogeneous and non-diploid or chromatin heterogeneous and diploid (CHO and ND or CHE and D), and chromatin heterogeneous and non-diploid (CHE and ND) among patients with high-risk stage II colon cancer.

According to the length of DFS, the combination of nucleotyping and DNA ploidy was divided into three groups, and found that CHO and diploid patients (low-risk group) had the highest 5-year DFS [89.7% (95% CI: 83.1–96.2%)] of the three groups, while the CHO and non-diploid or CHE and diploid patients had intermediate 5-year DFS [87.9% (95% CI: 79.8–96.0%)], referred to as medium-risk group. The HR was 1.845 (95% CI: 0.777–4.382). The CHE and non-diploid patients had the lowest 5-year DFS [71.4% (95% CI: 55.7–87.2%)], which is referred to as the high-risk group. The HR was 4.632 (95% CI: 1.995–10.752). The combination of nucleotyping and DNA ploidy was statistically significant for DFS (*P* < 0.001) (Table 2, Fig. 3 and Supplementary Table 2).

In terms of OS, nucleotyping and age were found to be significant factors in univariate analysis (*P* = 0.001 and *P* = 0.034, respectively). Stroma and other risk factors for stage II colon cancer were not significant predictors of OS. There was statistical difference in the combination of nucleotyping and DNA ploidy and the combination of nucleotyping and stroma when OS was used as the endpoint. However, there was no statistical difference in the combination of DNA ploidy and stroma (Table 2).

### Multivariable analyses of prognostic factors for DFS

In a multivariable model, nucleotyping, DNA ploidy, stroma, the combination of nucleotyping and stroma, the combination of DNA ploidy and stroma and the combination of nucleotyping and DNA ploidy were used as independent variables, respectively. Age and pathological T-stage were adjusted to establish multivariable analysis models.

After a correction for multiple comparisons, we found that nucleotyping and the combination of nucleotyping and DNA ploidy can be used as effective factors for DFS of high-risk stage II colon cancer.

In multivariable analysis model with the combination of nucleotyping and DNA ploidy as the independent variable, the dominant contributory factors on DFS was the combination of nucleotyping and DNA ploidy [HR 4.439 (95% CI: 1.909–10.319), *P* = 0.001] for the high-risk group vs. low-risk group. In multivariable analysis model with nucleotyping as the independent variable, the dominant contributory factor for DFS was nucleotyping [HR 3.309 (95% CI: 1.668–6.564), *P* = 0.001] for the CHE vs. CHO (Table 3).

**Table 3:** The value of independent variables in different Cox proportional hazards models as predictors of disease-free survival in high-risk II colon cancer.

Independent variables in different Cox proportional hazards models	HR (95% CI)	*P*-value
Stroma^a^		0.186
Low stroma	1	
High stroma	1.657 (0.784–3.520)	
DNA ploidy^a^		0.015
Diploid	1	
Non-diploid	2.585 (1.206–5.540)	
Nucleotyping^a^		0.001
Chromatin homogeneous	1	
Chromatin heterogeneous	3.309 (1.668–6.564)	
DNA ploidy and nucleotyping^a^		0.001
Diploid and chromatin homogeneous	1	
Diploid and chromatin heterogeneous or non-diploid and chromatin homogeneous	1.647 (0.700–4.002)	
Non-diploid and chromatin heterogeneous	4.439 (1.909–10.319)	
Stroma and nucleotyping^a^		0.008
Chromatin homogeneous and low stroma	1	
Chromatin homogeneous and high stroma or chromatin heterogeneous and low stroma	2.002 (0.952–4.211)	
Chromatin heterogeneous and high stroma	4.319 (1.655–11.268)	
Stroma and DNA ploidy^a^		0.024
Diploid and low stroma	1	
Diploid and high stroma or non-diploid and low stroma	1.818 (0.786–4.205)	
Non-diploid and high stroma	3.764 (1.442–9.852)	

A two-sided *p*-value of less than 0.008 was considered statistically significant.

^a^Adjusted age and pathological T-stage.

## Discussion

Although tumour-node-metastasis is currently the best prognostic factor for CRC, it still can not properly risk-stratify all patients, especially for stage II colon cancer patients.^2^ Whether patients with high-risk stage II colon cancer can benefit from adjuvant therapy is still controversial, suggesting tumour biological heterogeneity in these patients.^3,30^ A previous study has shown that chemotherapy improves survival in stage II colorectal cancer, although the absolute improvements are only 3.6%.^31^ Therefore, we sought to identify more accurate prognostic factors to further classify patients with high-risk stage II colon cancer. Our study demonstrated that nucleotyping was the dominant prognostic factor in high-risk patients with stage II colon cancer, whether in univariate or multivariable analyses. In high-risk stage II colon cancer patients, the DFS of CHE patients was significantly shorter than that of CHO patients. For CHO patients, our research showed that their 5-year DFS rate was high (88.8%). These results lead to the conclusion that nucleotyping is an independent prognostic factor in high-risk stage II colon cancer. Based on these evidences, it is necessary to further stratify those patients for the purpose of personalised treatment in the future.

Tumour progression is accompanied by genomic and epigenetic changes that make tumours progressively aggressive.^32,33^ These changes alter the nuclei in multiple ways: including the size of the nucleus, the density of DNA, and the structure of chromatin. Nucleotyping is a quantitative analysis of the degree of nuclear disorder of tumours by integrating the three types of changes in the tumour cell nuclei through artificial intelligence, in order to ascertain the degree of malignancy of tumour cells in different patients. A pan-cancer study has shown that nucleotyping can be a prognostic factor for many tumours, including stage I or II colorectal cancer. Cancer-specific survival rates in patients with CHE are lower than that of patients with CHO.^20^ Our research produced similar results, since we found that nucleotyping could predict the prognosis of patients with high-risk stage II colon cancer.

To our knowledge, this study was the first external and independently performed evaluation of nucleotyping by a completely different team than the original Kleppe A team.^20^ The multivariable analysis model displayed with nucleotyping as independent variable could predict recurrence and metastasis in high-risk stage II colon cancer patients. Therefore, we believe nucleotyping can further stratify existing high-risk stage II colon cancer patients, providing a more accurate basis for guiding clinical personalised medicine.

Consistent with previous studies, pathological T-stage had an impact on the prognosis of patients in our study.^29,34^ Previous studies have shown that in multivariable analysis, increased age and the absence of adjuvant chemotherapy are risk factors for stage II colon cancer.^35,36^ Different from these studies, our study showed that adjuvant chemotherapy had no significant effect on survival or recurrence and metastasis of cancer in patients, while the overall survival rate of older patients was higher than that of younger patients. This may be attribute to the fact that our study subjects were high-risk stage II colon cancer patients, with the median age was younger than that of study mentioned above.^36^

This study was consistent with another study that found that DNA ploidy was negatively correlated with dMMR status.^14^ This may be attributed to methylation of many promoters being associated with tumour diploidy and dMMR.^37^ Nucleotyping markers can reflect the degree of chromosomal disorder, including information about the chromatin structure, which can explain the correlation between nucleotyping and DNA ploidy.

Sheltzer et al.^38^ reported that aneuploid genomic instability may contribute to aggressive growth of advanced malignant tumours with complex karyotypes. Although the mechanisms by which non-diploid tumours affect the prognosis of patients has not been well elucidated, previous studies have shown that non-diploid tumours promote the development of tumours,^39^ and whole genome doubling promotes resistance to a broad spectrum of chemotherapeutic drugs and can also lead to genomic instability.^40^ Non-diploid tumour has been proven a statistically significant predictor of poor prognosis of many cancers, including CRC.^13,14,41^ The results of our univariate analysis suggested that, similar to previous results, there was no significant difference in overall survival between diploid and non-diploid patients, this might attribute to a small sample size in our study. However, the DFS of diploid patients was significantly longer than that of non-diploid patients.^14,29^

Previous studies have confirmed that patients with stroma-high stage II colorectal cancer have a poor prognosis.^35,36,42–46^ One study showed that patients with stroma-high tumours in CRC had worse OS compared to patients with stroma-low tumours. Alternatively, in analysis of DFS, no significant differences were observed between patients with stroma-high and stroma-low tumours.^42^ Our results showed that the prognosis of the high-stroma group was worse than that of the low-stroma group, regardless of whether OS or DFS was used as the study endpoint. However, the difference was not statistically significant, contrary to previous studies, but this might be due to the small sample size and low power calculation (1 − β = 0.184). Expanding larger the sample size is required to accurately analyse the prognostic value of stroma. For stroma analysis, as was described by Danielsen et al.,^29^ we studied tumour-enriched areas and evaluate all tumour areas on whole scanned images, rather than studied the areas with the deepest part of the tumour and to use a microscope with an area the size of a ×10 objective to select tumour areas. Besides, some patients did not receive adjuvant therapy because they were too old or for financial reason, this may affect the prognosis. All of the above reasons may lead to different results from previous studies.

The present study was limited by single-centre design with small sample size, sampling error and short follow-up time, criteria for assessment, and different adjuvant chemotherapy regiments used. Fewer cases of recurrence, metastasis and death may reduce the quality of statistical analysis. Future research will be directed at following up and expanding the sample size to further determine the reliability of the results herein.

## Supplementary information

Supplemental tables

## Data Availability

All data not included in this published article are available upon reasonable request. Supplementary information is available on the British Journal of Cancer’s website.
