# Supplementary material for: Prognostic value of nucleotyping, DNA ploidy and stroma in high-risk stage II colon cancer
Source: Br J Cancer. 2020 Jul 6;123(6):973–81. doi: 10.1038/s41416-020-0974-8 (PMC7492254; doi:10.1038/s41416-020-0974-8)
Supplement: Supplementary file 1 — Supplemental tables [file 41416_2020_974_MOESM1_ESM.doc]

Supplemental Information

**Prognostic value of nucleotyping, DNA ploidy and stroma in high-risk stage II colon cancer**

Lujing Yang, Pengju Chen, Li Zhang, Lin Wang, Tingting Sun, Lixin Zhou, Zhongwu Li, Aiwen Wu

**Supplementary table 1** Coefficient between nucleotyping and ploidy

| **Variable** | **Chromatin homogeneous** | **Chromatin heterogeneous** | **Coefficient** | **P-value** |
| --- | --- | --- | --- | --- |
| **DNA ploidy** |  |  | 0.743 | <0.001 |
| Diploid | 87(57.2%) | 1(2.8%) |  |  |
| Non-diploid | 65(42.8%) | 35(97.2%) |  |  |

Data are number (%), unless otherwise indicated.

**Supplementary table 2** five-year overall survival and five-years disease-free for different variable groups

| **Variable** | **n** | **5-year OS, %** | **95%CI, %** | **5-year DFS, %** | **95%CI, %** |
| --- | --- | --- | --- | --- | --- |
| **Age** |  |  |  |  |  |
| **≤**63 | 90 | 84.4 | 76.8-92.1 | 82.2 | 74.2-90.2 |
| >63 | 98 | 93.9 | 89.1-98.7 | 88.8 | 82.4-95.1 |
| **Lymph nodes sampling** |  |  |  |  |  |
| >12 | 152 | 89.5 | 84.5-94.4 | 85.5 | 79.9-91.2 |
| <12 | 36 | 88.9 | 78.1-99.7 | 86.1 | 74.2-97.9 |
| **Histological grade** |  |  |  |  |  |
| Well differentiated | 7 | 71.4 | 26.3-100.0 | 71.4 | 26.3-100.0 |
| Moderately differentiated | 123 | 89.4 | 83.9-94.9 | 83.7 | 77.1-90.3 |
| Poorly differentiated | 55 | 90.9 | 83.0-98.8 | 90.9 | 83.0-98.8 |
| [mucinous](http://www.baidu.com/link?url=rCI8W6-xjI0fs5P61AWsVdrqTNbcTJsG6R_1QpFe4r7MA-a9t9xhb34064vMV4OJDLYtUXUQFkDpKnVlJA2POkXfNp34N-slvR3YJ6o1u4FEs-IV2F-VEREg1f5O3J4R) | 3 | 100.0 | - | 100.0 | - |
| **Vascular or perineural invasion** |  |  |  |  |  |
| Yes | 55 | 87.3 | 78.2-96.4 | 81.8 | 71.3-92.3 |
| No | 133 | 90.2 | 85.1-95.3 | 87.2 | 81.5-93.0 |
| **pTstage** |  |  |  |  |  |
| pT3 | 125 | 92.8 | 88.2-97.4 | 89.8 | 84.2-95.0 |
| pT4 | 63 | 82.5 | 72.9-92.2 | 77.8 | 67.2-88.3 |
| **Intestinal occlusion or perforation** |  |  |  |  |  |
| Yes | 37 | 86.5 | 74.9-98.0 | 83.8 | 71.3-96.2 |
| No | 151 | 90.1 | 85.2-94.9 | 86.1 | 80.5-91.7 |
| **Mismatch repair status** |  |  |  |  |  |
| pMMR | 153 | 86.3 | 80.2-92.4 | 82.3 | 75.4-89.1 |
| dMMR | 64 | 95.3 | 90.0-100.6. | 92.2 | 85.4-98.9 |
| **Adjuvant chemotherapy** |  |  |  |  |  |
| No | 81 | 87.7 | 80.3-95.0 | 84.0 | 75.8-92.1 |
| Yes | 107 | 90.7 | 85.1-96.3 | 86.9 | 80.4-93.4 |
| **DNA ploidy** |  |  |  |  |  |
| Diploid | 88 | 90.9 | 84.8-97.0 | 89.8 | 83.3-96.2 |
| Non-diploid | 100 | 88.0 | 81.5-94.5 | 82.0 | 74.3-89.7 |
| **Stroma** |  |  |  |  |  |
| Low-stroma | 153 | 90.2 | 85.4-95.0 | 87.6 | 82.3-92.9 |
| High-stroma | 35 | 85.7 | 73.5-97.9 | 77.1 | 62.5-91.8 |
| **Nucleotyping** |  |  |  |  |  |
| Chromatin homogeneous | 152 | 91.4 | 86.9-95.9 | 88.8 | 83.7-93.9 |
| Chromatin heterogeneous | 36 | 80.6 | 67.0-94.1 | 72.2 | 56.9-87.6 |
| **DNA ploidy and Stroma** |  |  |  |  |  |
| Diploid and Low-stroma | 77 | 90.9 | 84.3-97.5 | 89.6 | 82.6-96.6 |
| Diploid and High-stroma or Non-diploid and Low-stroma | 87 | 89.7 | 83.1-96.2 | 86.2 | 78.8-93.6 |
| Non-diploid and High-stroma | 24 | 83.3 | 67.3-99.4 | 70.8 | 51.2-90.4 |
| **Nucleotyping and Stroma** |  |  |  |  |  |
| Chromatin homogeneous and Low-stroma | 130 | 92.3 | 87.7-97.0 | 89.2 | 83.8-94.6 |
| Chromatin homogeneous and High-stroma or Chromatin heterogeneous and Low-stroma | 45 | 82.2 | 70.6-93.8 | 82.2 | 70.6-93.8 |
| Chromatin heterogeneous and High-stroma | 13 | 84.6 | 62.9-107.3 | 61.5 | 30.9-92.1 |
| **DNA ploidy and Nucleotyping** |  |  |  |  |  |
| Diploid and Chromatin homogeneous | 87 | 90.8 | 84.6-97.0 | 89.7 | 83.1-96.2 |
| Diploid and Chromatin heterogeneous or Non-diploid and Chromatin homogeneous | 66 | 92.4 | 85.9-99.0 | 87.9 | 79.8-96.0 |
| Non-diploid and Chromatin heterogeneous | 35 | 80.0 | 66.1-93.9 | 71.4 | 55.7-87.2 |

pMMR, mismatch repair proficient; dMMR, mismatch repair deficient; OS, overall survival; DFS, disease-free survival

**Supplementary table3** Coefficient between ploidy, stroma and nucleotyping and high-risk factors

| **Variable** | **Diploid** | **Non-diploid** | **ρ** | ***P*-value** | **Low-stroma** | **High-stroma** | **ρ** | ***P*-value** | **CHO** | **CHE** | **ρ** | **P-value** |
| --- | --- | --- | --- | --- | --- | --- | --- | --- | --- | --- | --- | --- |
| **Lymph nodes sampling** |  |  | 0.077 | 0.292 |  |  | 0.115 | 0.118 |  |  | 0.107 | 0.145 |
| >12 | 74(84.1%) | 78(78.0%) |  |  | 127(83.0%) | 25(71.4%) |  |  | 126(82.9%) | 26(72.2%) |  |  |
| <12 | 14(15.9%) | 22(22.0%) |  |  | 26(17.0%) | 10(28.6%) |  |  | 26(17.1%) | 10(27.8%) |  |  |
| **Histological grade** |  |  | -0.215 | 0.005 |  |  | -0.130 | 0.075 |  |  | -0. 079 | 0.278 |
| Well differentiated | 3(3.4%) | 4(4.0%) |  |  | 5(3.3%) | 2(5.7%) |  |  | 7(4.6%) | 0(0.0%) |  |  |
| Moderately differentiated | 48(54.5%) | 75(75.0%) |  |  | 96(62.7%) | 27(77.1%) |  |  | 94(61.8%) | 29(80.5%) |  |  |
| Poorly differentiated | 35(39.8%) | 20(20.0%) |  |  | 51(33.3%) | 4(11.4%) |  |  | 49(32.2%) | 6(16.7%) |  |  |
| [Mucinous](http://www.baidu.com/link?url=rCI8W6-xjI0fs5P61AWsVdrqTNbcTJsG6R_1QpFe4r7MA-a9t9xhb34064vMV4OJDLYtUXUQFkDpKnVlJA2POkXfNp34N-slvR3YJ6o1u4FEs-IV2F-VEREg1f5O3J4R) | 2(2.3%) | 1(1.0%) |  |  | 1(0.7%) | 2(5.8%) |  |  | 2(1.4%) | 1(2.8%) |  |  |
| **Vascular or perineural invasion** |  |  | 0.017 | 0.812 |  |  | -0.127 | 0.082 |  |  | 0.014 | 0.850 |
| No | 63(71.6%) | 70(70.0%) |  |  | 104(68.0%) | 29(82.9%) |  |  | 108(71.1%) | 25(69.4%) |  |  |
| Yes | 25(28.4%) | 30(30.0%) |  |  | 49(32.0%) | 6(17.1%) |  |  | 44(28.9%) | 11(30.6%) |  |  |
| **pTstage** |  |  | 0.011 | 0.880 |  |  | 0.153 | 0.037 |  |  | 0.084 | 0.251 |
| pT3 | 59(67.0%) | 66(66.0%) |  |  | 107(69.9%) | 18(51.4%) |  |  | 104(68.4%) | 21(58.3%) |  |  |
| pT4 | 29(33.0%) | 34(34.0%) |  |  | 46(30.1%) | 17(48.6%) |  |  | 48(31.6%) | 15(41.7%) |  |  |
| **Intestinal occlusion or perforation** |  |  | 0.116 | 0.114 |  |  | 0.176 | 0.016 |  |  | -0.037 | 0.615 |
| No | 75(85.2%) | 76(76.0%) |  |  | 128(83.7%) | 23(65.7%) |  |  | 121(79.6%) | 30(83.3%) |  |  |
| Yes | 13(14.8%) | 24 (24.0%) |  |  | 25(16.3%) | 12(34.3%) |  |  | 31(20.4%) | 6(16.7%) |  |  |
| **Mismatch repair status** |  |  | -0.237 | 0.001 |  |  | -0.109 | 0.135 |  |  | 0.223 | 0.002 |
| pMMR | 48(54.5%) | 77(77.0%) |  |  | 99(64.7%) | 26(74.3%) |  |  | 96(63.2%) | 29(80.6%) |  |  |
| dMMR | 40(45.5%) | 23(23.0%) |  |  | 54(35.3%) | 9(25.7%) |  |  | 56(36.8%) | 7(19.4%) |  |  |

Data are number (%), unless otherwise indicated. CHE, chromatin heterogeneous; CHO, chromatin homogeneous
